# Feminisation of the health workforce and wage conditions of health professions: an exploratory analysis

**DOI:** 10.1186/s12960-019-0406-0

**Published:** 2019-10-17

**Authors:** Geordan Shannon, Nicole Minckas, Des Tan, Hassan Haghparast-Bidgoli, Neha Batura, Jenevieve Mannell

**Affiliations:** 1 Centre for Gender and Global Health, Institute for Global Health, University College London, 3rd floor, Institute of Child Health, 30 Guilford Street, London, WC1N 1EH UK; 2 STEMA, Institute for Global Health, University College London, 3rd floor, Institute of Child Health, 30 Guilford Street, London, WC1N 1EH UK; 3 Centre for Global Health Economics, Institute for Global Health, University College London, 3rd floor, Institute of Child Health, 30 Guilford Street, London, WC1N 1EH UK

**Keywords:** Health workforce, Wage conditions, Gender, Wage gap, Inequalities, Feminist economics

## Abstract

**Background:**

The feminisation of the global health workforce presents a unique challenge for human resource policy and health sector reform which requires an explicit gender focus. Relatively little is known about changes in the gender composition of the health workforce and its impact on drivers of global health workforce dynamics such as wage conditions. In this article, we use a gender analysis to explore if the feminisation of the global health workforce leads to a deterioration of wage conditions in health.

**Methods:**

We performed an exploratory, time series analysis of gender disaggregated *WageIndicator* data. We explored global gender trends, wage gaps and wage conditions over time in selected health occupations. We analysed a sample of 25 countries over 9 years between 2006 and 2014, containing data from 970,894 individuals, with 79,633 participants working in health occupations (48,282 of which reported wage data). We reported by year, country income level and health occupation grouping.

**Results:**

The health workforce is feminising, particularly in lower- and upper-middle-income countries. This was associated with a wage gap for women of 26 to 36% less than men, which increased over time. In lower- and upper-middle-income countries, an increasing proportion of women in the health workforce was associated with an increasing gender wage gap and decreasing wage conditions. The gender wage gap was pronounced in both clinical and allied health professions and over lower-middle-, upper-middle- and high-income countries, although the largest gender wage gaps were seen in allied healthcare occupations in lower-middle-income countries.

**Conclusion:**

These results, if a true reflection of the global health workforce, have significant implications for health policy and planning and highlight tensions between current, purely economic, framing of health workforce dynamics and the need for more extensive gender analysis. They also highlight the value of a more nuanced approach to health workforce planning that is gender sensitive, specific to countries’ levels of development, and considers specific health occupations.

## Introduction

The feminisation of the health workforce—the movement of women into occupations where they were formally under-represented [1]– is a phenomenon that has been extensively documented in global health research [1–13]. In medicine, women have moved from exclusion from the profession to the majority of medical graduates in many countries around the world [2, 3]. Feminisation of the medical profession has been recorded in countries as diverse as Bangladesh [4], Canada [5], Cape Verde [6], Guinea Bissau [6], Israel [7], Mozambique [6], Oman [8], the UK [3] and the US [9]. In dentistry, the proportion of women is projected to increase to 28% globally by 2030 [10]. Women now comprise approximately 75% of the global health workforce [11], and over 90% of nursing and midwifery professions [12]. Despite the shifting gender balance of the health workforce, women still tend to belong to lower cadres of health workers [11, 13], are under-represented in positions of leadership [12, 14], are over-represented in unskilled and unpaid work [13], and earn less than men [11, 12].

These dynamics present a challenge for human resource policy and health sector reform. With a predicted shortfall of over 18 million health workers by 2030 to achieve universal health coverage (UHC), investing in human resources for health is an international priority [15]^.^

Despite this, relatively little is known about the impact of the feminisation of global health on core drivers of health workforce dynamics, such as wage conditions. Wages are widely regarded as a factor that influence job satisfaction and may drive the “…migration of healthcare professionals within and across countries” [16] and comprise a major component of national government health expenditure [17]. Discrete, cross-sectional research has suggested that gender is linked to wage inequalities in health research [18], medicine [19], and even in traditionally women-dominated professions such as nursing [20, 21]. In a 20-country study, a cross-sectional analysis of 16 occupations demonstrated that a 1% increase in the proportion of women in a certain occupation was associated with an 8% decrease in wage rank compared to other healthcare occupations [22]. With a body of research establishing gender wage gaps in the health workforce, there is a need to explore data on wage trends *over time* from a gender perspective and to position this in relation to the feminisation of the health workforce.

Research on wage conditions and the feminisation of the global health workforce has been limited by lack of internationally comparable, gender-disaggregated wage data that contain sufficiently detailed information about health sector occupations and their corresponding wages. Many countries have limited ability to report healthcare wages due to infrastructural barriers [16]. International Labour Organization (ILO) and Organisation for Economic Cooperation and Development (OECD) data often report highly aggregated occupational levels or do not present gender-disaggregated information [22, 23]. Owing to these limitations, critical, evidence-based discussions about gender, the health workforce and wage condition trends are limited.

In this article, we present the trends of the global health workforce with an explicit focus on gender and examine if and how these trends are associated with changing wage conditions over time. We perform an exploratory time series analysis of gender disaggregated data from the *WageIndicator* dataset between 2006 and 2014. Our proposed strategy builds from the methodology proposed by Tijdens et al., who extracted age, gender, education, occupation and salary data over 20 countries and presented a pooled analysis [22]. Here, we use an exploratory, time series analysis to examine differences in participation and remuneration over time to extend our understandings of gender trends in the global health workforce and its impact on wage conditions.

## Methods

### Gender analysis and the gender division of labour in healthcare

Gender refers to the “socially constructed norms that impose and determine roles, relationships and positional power for all people across their lifetime. Gender interacts with sex, the biological and physical characteristics that define women, men and those with intersex identities” [24]. Gender can be conceptualised as a system of social stratification that determines interpersonal interactions and shapes access to resources and power [24–26]. As such, gender is a critical factor in determining the position of women, men and gender-diverse people in the health workforce and their subjective experiences [13].

Health systems reflect the social, political and economic contexts they operate in, including gendered social norms [27, 28]. A gender analysis in health systems research involves asking questions about the gendered nature of research, programmes or policies and their impact [27, 29]. Gender analysis can be incorporated into research on the health workforce by sex disaggregation of data, using a feminist or gender lens in the analysis of data, or reflecting on power relations in health systems and how these may be transformed [30].

In this paper, we look at the gendered division of labour to inform our particular gender analysis. The gendered division of labour refers to the way work (paid and unpaid) is divided between men and women according to their gender [31]. The health workforce has historically been subject to distinct gender divisions, where professions such as medicine and dentistry were dominated by men and caregiving or support roles were seen as women’s jobs [32]. Although the gender division of labour in healthcare is changing, legacies of gender stereotypes—replicated throughout the health workforce—serve as significant restrictions to healthcare labour roles. For example, caregiving work, often performed by women, remains under-supported and under-valued in current-day health systems [13, 23].

**Fig. 1:**
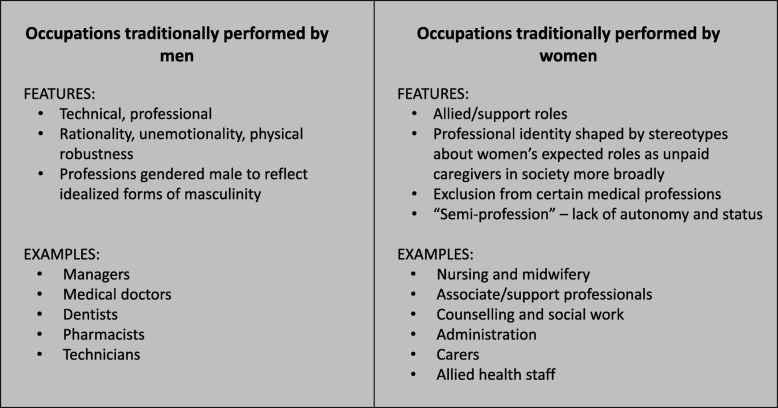
Conceptual framework—historical gender division of the health workforce. The gendered nature of the health workforce has been shaped by broader gender norms. See references [31–37]

Figure 1 demonstrates a basic conceptual framework to support our analysis. Gender divisions in the health workforce have been shaped by broader stereotypes about men’s and women’s gender roles in society [32–34]. Professions such as medicine and dentistry were “gendered male” [32] to reflect idealised forms of masculinity such as rationality, unemotionality, physical robustness, whereas professions “gendered female” were shaped by stereotypes about women’s expected roles as unpaid caregivers in society more broadly [35, 36]. Historically, women were excluded from the right to practice in certain medical professions [32], and women’s health work was considered a “semi-profession” because of the lack of autonomy and status [32, 37].

### Gender and wage data

*WageIndicator* is a Dutch online platform containing information about national labour markets, including salary checks, labour laws and minimum wage information. The website is visited over 200,000 times per month by students, job-seekers, employees and self-employed persons around the world [22, 38]. Visitors to the site participate in a voluntary questionnaire regarding their occupation and wages. Around 5% of visitors—more than 1 million individuals—have completed the survey. The questionnaire is comparable across countries, presented in the national language(s) and adapted to local contexts [22]. Survey questions, presented in detail by Tijdens et al., contain self-reported information on gender (“are you a woman or a man?”), sociodemographic characteristics, country, occupation, wages and other work-related details [22]. We were granted access to data for free for the purpose of academic research from the IZA, Germany, at http://idsc.iza.org/?page=27&stid=1025 [39].

The drawbacks of web-based survey data such as WageIndicator—including self-selection and reporting bias—have been detailed elsewhere [40, 41] and will be discussed in depth in the limitations, below. Previous studies show that *WageIndicator* data deviated from national reference samples over gender, age and level of education [41]. In particular, survey participants over 40 years of age were under-represented, possibly due to lower levels of computer literacy in older age groups [22]. Drawing on previous strategies used, we applied a simple proportional weighting by country to adjust our data to ILO global Economically Active Population Estimates and Projections (EAPEP) distributions [42]. Given these limitations, the data should be considered exploratory rather than representative [22]. However, to our knowledge, *WageIndicator* data is currently the only resource that contains both gender-disaggregated data and sufficiently detailed information about health sector occupations and trends in wages over time.

### Country selection and grouping

We included countries that contained information from over 1000 participants and excluded countries that had more than two consecutive years of missing data, or countries that demonstrated significant attrition (> 80% per year) in survey response over time. We narrowed our timeframe between 2006 and 2014 due to poor survey response before 2006 and lack of information after 2015. This provided a sample of 25 countries over 9 years containing 1,798,412 observations, with wage-related information available for 970,894 of these observations.

Given the restricted size of the dataset, and risk of sampling error due to small sample sizes in some country-year cells, we were not able to present results by individual countries. Instead, we grouped countries by their World Bank classification [43] for 2017, according to gross national income (GNI) per capita. Table 1 presents a summary of the countries included in the analysis, grouped by income classification level.

**Table 1:** Summary of country groupings according to World Bank income classification, 2017

Country	Survey participants reporting wage-related information	
	Total workforce (*n*)	Health workforce (*n*)
Lower-middle-income countries (LMIC): GNI per capita $1 006 to $3 955		
Angola	924	35
India	31 382	377
Indonesia	16 703	315
Ukraine	34 803	1 567
Vietnam	4 055	14
Sub-total	87 867	2 308
Upper-middle-income countries (UMIC): GNI per capita $3 956 to $12 235		
Argentina	56 212	1735
Azerbaijan	3 460	93
Belarus	46 849	1 663
Brazil	74 160	2 907
Colombia	7 614	392
Kazakhstan	23 194	676
Mexico	26 111	762
Paraguay	4 475	96
Russian Federation	14 262	632
South Africa	35 856	774
Sub-total	292 193	9 730
High-income countries (HIC): GNI per capita $12 236 or more		
Belgium	41 050	2 901
Chile	9 413	439
Czech Republic	18 695	1 117
Finland	29 184	2 233
Germany	185 498	12 465
Hungary	13 972	640
Netherlands	207 929	12 227
Spain	29 637	1 319
United Kingdom	46 393	2 233
United States	9 063	670
Sub-total	590 834	36 244
Total	970 894	48 282

### Gender, occupation and health worker wages

We defined health occupations according to the WHO Global Atlas of the Health Workforce international classification of health workers, based on certain four-digit identifying codes derived from the International Standard Classification of Occupations, 2008 revision (ISCO-08) [44]. The self-identified occupations reported by *WageIndicator* are coded according to ISCO-08 classifications [22]. This process has yielded accurate results that have been validated internationally [45].

We examined 37 health occupations, coded to the four-digit ISCO-08 level. We categorised health occupations into 15 professional groups representing healthcare managers, medical doctors, pharmacists, dentists, technicians, nurses and midwives, community health workers, health associate professionals, administration, carers, traditional medical practitioners and allied health staff (Table 2). Using the conceptual framework outlined in Fig. 1, we further grouped health occupations by whether they were traditionally dominated by men (clinical or technical occupations such as medicine) or dominated by women (allied or support professions, such as nursing and carers), in order to capture the gendered division of labour, and how this may have changed over time. These groupings can be found in Table 2, below.

**Table 2:** Health occupation groupings by the ISCO-08 four-digit classification system

Clinical, technical or managerial occupations	Allied, caregiving or associate occupations	
Traditionally male-dominated	Traditionally female-dominated	
1. Health service managers	6. Nursing ad midwifery professionals	12. Carers in health services
1 342 health service manager	2 221 nursing professionals	5 321 healthcare assistance
1 343 aged care service manager	2 222 midwifery professionals	5 322 home-based personal care workers
2. Medical doctors	7. Nursing and midwifery associate professionals	5 329 personal care workers in health services not elsewhere classified
2 211 generalist medical practitioners	3 221 nursing associate professionals	13. Traditional and complementary medicine professionals
2 212 specialist medical practitioners	3 222 midwifery associate professionals	2 230 traditional and complementary medicine professionals
3. Dentists	8. Community health workers	3 230 traditional and complementary medicine associate professionals
2 261 dentists	3 253 community health workers	14. Paramedical practitioners
4. Pharmacists	9. Other health associate professionals	2 240 paramedical practitioners
2 262 pharmacists	3 251 dental assistants and therapists	15. Allied health staff
5. Medical and pharmaceutical technicians	3 254 dispensing opticians	2 263 environmental and occupational health and hygiene professionals
3 211 medical imaging and therapeutic equipment technicians	3 255 physiotherapy technicians and assistants	2 264 physiotherapists
3 212 medical and pathology laboratory technicians	3 256 medical assistants	2 265 dieticians and nutritionists
3 213 pharmaceutical technicians and assistants	3 257 environmental and occupational health inspectors and associates	2 266 audiologists and speech therapists
3 214 medical and dental prosthetic technicians	3 258 ambulance workers	2 267 optometrists and ophthalmic opticians
	3 259 health associate professionals not elsewhere classified	2 269 health professionals not elsewhere classified
	10. Counselling and social work	
	2 635 counselling and social work	
	11. Administration and medical records	
	3 344 medical secretary	
	3 252 medical records and health information technicians	

We then extracted wage information for each individual in our dataset where it was available. *WageIndicator* data contains information on self-reported wages, transformed to gross reported wages per hour, converted to an international dollar using a purchasing power parity (PPP) conversion factor for each country. PPP is calculated based on an exchange rate that compares and equalises a basket of goods and services between countries [42]. We excluded the top and bottom 0.05% of observations (*n* = 80), as these may be outliers due to erroneous self-reported responses. Restricting the analysis to health occupations resulted in 79,633 remaining observations, of which 48,282 reported wage data.

**Fig. 2:**
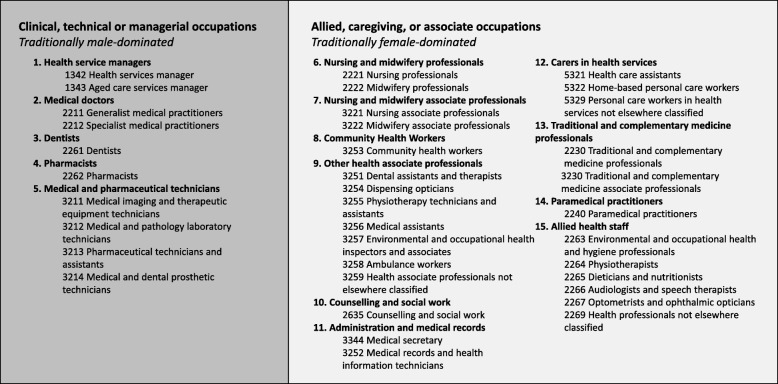
Selection and analysis of WageIndicator data

A summary of our data selection process is available in Fig. 2. The final dataset contained information from a total of 1,798,412 individuals from 25 countries; we analysed data from 970,894 participants in the general workforce who reported gender and wage data, and 79,633 participants in the health workforce (of which 48,282 participants reported wage data) between 2006 and 2014.

### Analysis

We performed an exploratory, descriptive analysis of country groups (described in Table 1) and occupation groups (described in Table 2) by years between 2006 and 2014. Data was insufficient for analysis prior to 2006 or after 2015. We examined gender trends in participation, remuneration and health worker wage conditions.

To examine gender trends in participation, we calculated the unadjusted *gender ratio* (proportion of women workers compared to total workers) by country group and year, and presented this information by the general workforce, the overall health workforce and the gendered grouping of health occupations (whether the occupation was traditionally dominated by men or women, Fig. 1 and Table 2).

To examine gender trends in remuneration, we calculated the *gender wage gap* as the difference between average gross hourly earnings of men and average gross hourly earnings of women expressed as a proportion of average gross hourly earnings of men [46]. This was calculated by country group and year, over the general workforce, the overall health workforce and whether the health occupation was traditionally clinical/technical (dominated by men, higher paid) or allied/caregiving (dominated by women, lower paid).

To examine wage conditions of the health workforce, we calculated the average general workforce wage, defined as the mean reported salary of all survey participants (healthcare and non-healthcare professions) by country and year. We then calculated the ratio between the pooled health occupation wage and the average national wage. We define this as the *healthcare occupation wage ratio*.

To examine temporal changes in the gender wage gap across health occupation and country groups, we calculated the average annual percentage change (AAPC) for each country grouping and health occupation group using the Jointpoint Regression Program V.3.5.4. Annual percentage change (APC) is calculated using weighted least squares regression. AAPC represents a summary measure of the APC trend over a pre-specified interval of time and is computed by taking the weighted average of annual changes over a period of multiple years. The Jointpoint Regression Program uses a Monte Carlo Permutation method as a test of significance in trend. This approach at its application has been described in more depth by the National Cancer Institute [47] and has been applied in epidemiological research [48].

We performed additional descriptive analysis in Python and Excel.

## Results

Within the population who reported wage data, there was a gender balance of 43.4% men and 56.6% women (see Supplementary Demographic Table). Women’s participation in the survey varied from 35.6% in Angola (corresponding to 64.4% participation by men) to 83.3% in India (corresponding to 16.7% participation by men). Reported ages ranged from 7 to 81 years, with the majority of participants between 20 and 39 years at the time of survey completion. In those who completed the survey, 44.7% of participants reported a high level of education (International Standard Classification of Education, ISCED, level 5-6); 30.2% reported a medium level of education (ISCED level 3-4); 18.5% reported a low level of education (ISCED level 0-2); and information on education levels was missing from 6.6% participants’ responses. Although we were unable to further disaggregate by education or age in our analysis, we assume that elements of education are, to some degree, subsumed in the profession (in that some occupational groups reflect necessary prerequisite education).

Table 3 provides a summary of the results of our analysis, including the gender ratio, the gender wage gap and the healthcare occupation wage ratio, as well as the AAPC trend for gender wage ratios and gender wage gaps. We present results of each stage of analysis, below.

**Table 3:** Gender ratio, gender wage gap and healthcare occupation wage ratio by country group and year, with average annual percentage change (AAPC)

Results	Year of analysis									AAPC
	2006	2007	2008	2009	2010	2011	2012	2013	2014	
Gender ratio (%, *n*)										
Support and allied healthcare										
HIC	0.80 (5 573)	0.78 (4 904)	0.79 (7 581)	0.79 (3 616)	0.82 (4 571)	0.82 (4 983)	0.83 (4 521)	0.82 (3 968)	0.82 (5 973)	0.7*
UMIC	0.57 (280)	0.70 (852)	0.68 (1 220)	0.66 (788)	0.71 (1 105)	0.81 (2 220)	0.81 (2 456)	0.80 (1 468)	0.80 (1 291)	3.9*
LMIC		0.67 (7)	0.67 (28)	0.47 (72)	0.53 (142)	0.70 (945)	0.72 (992)	0.70 (603)	0.72 (615)	2.8
Clinical and technical healthcare										
HIC	0.68 (1 162)	0.74 (763)	0.72 (1 317)	0.67 (912)	0.71 (756)	0.73 (924)	0.73 (741)	0.78 (598)	0.75 (930)	1.1
UMIC	0.50 (93)	0.45 (421)	0.54 (691)	0.42 (650)	0.57 (820)	0.63 (1 315)	0.68 (1 254)	0.68 (683)	0.70 (614)	5.8*
LMIC		0.36 (10)	0.33 (27)	0.27 (44)	0.37 (113)	0.57 (698)	0.62 (631)	0.54 (441)	0.57 (306)	4.5
All healthcare										
HIC	0.73 (6 735)	0.75 (5 667)	0.74 (8 898)	0.71 (4 628)	0.76 (5 327)	0.76 (5 907)	0.77 (5 262)	0.79 (4 566)	0.79 (6 903)	1.1*
UMIC	0.52 (373)	0.50 (1 273)	0.57 (1 911)	0.47 (1 438)	0.60 (1 925)	0.68 (3 535)	0.71 (3 710)	0.72 (2 151)	0.73 (1 905)	5.6*
LMIC		0.41 (17)	0.36 (55)	0.30 (116)	0.41 (255)	0.60 (1 643)	0.65 (1 623)	0.58 (1 044)	0.62 (921)	6.4
General workforce										
HIC	0.47 (137 408)	0.42 (109 434)	0.40 (178 940)	0.42 (87 713)	0.41 (90 844)	0.43 (100 617)	0.43 (84 755)	0.44 (75 334)	0.43 (101 321)	0
UMIC	0.40 (14 109)	0.42 (37 369)	0.45 (79 430)	0.36 (46 728)	0.44 (61 254)	0.49 (121 838)	0.53 (114 178)	0.53 (67 127)	0.54 (48 887)	4.3*
LMIC	0.20 (3 154)	0.24 (938)	0.19 (5 967)	0.23 (8 731)	0.22 (17 188)	0.41 (72 928)	0.44 (58 797)	0.39 (42 214)	0.41 (31 209)	11.8*
Gender wage gap (mean, standard deviation)										
Support and allied healthcare										
HIC	0.10 (0.14)	0.18 (0.26)	0.17 (0.26)	0.22 (0.28)	0.27 (0.46)	0.21 (0.30)	0.22 (0.33)	0.23 (0.35)	0.18 (0.39)	15.4
UMIC	0.14 (0.39)	− 0.01 (0.01)	− 0.11 (0.34)	0.05 (0.13)	0.33 (0.94)	0.21 (0.47)	0.39 (1.31)	0.18 (0.48)	0.36 (0.87)	14.9*
LMIC		0.95 (1.14)	0.80 (1.83)	0.17 (0.39)	0.73 (2.05)	0.61 (2.48)	0.51 (1.40)	0.80 (2.27)	0.21 (0.87)	4.8
Clinical and technical healthcare										
HIC	0.38 (0.51)	0.27 (0.42)	0.33 (0.49)	0.40 (0.50)	0.53 (0.81)	0.45 (0.74)	0.48 (0.61)	0.27 (0.44)	0.23 (0.45)	0.6
UMIC	− 0.03 (0.08)	0.25 (0.58)	0.30 (0.62)	0.24 (0.58)	0.36 (0.83)	0.26 (0.52)	0.17 (0.49)	0.50 (1.21)	0.54 (1.20)	11.3*
LMIC		0.81 (1.32)	− 1.01 (1.55)	0.23 (0.34)	0.40 (1.10)	0.24 (0.81)	0.36 (0.73)	0.51 (1.52)	0.43 (1.44)	3.9
All healthcare										
HIC	0.26 (0.38)	0.24 (0.36)	0.25 (0.39)	0.29 (0.38)	0.38 (0.63)	0.33 (0.50)	0.34 (0.48)	0.26 (0.41)	0.24 (0.51)	1
UMIC	0.09 (0.25)	0.15 (0.38)	0.12 (0.32)	0.23 (0.58)	0.37 (0.97)	0.26 (0.56)	0.31 (1.00)	0.36 (1.05)	0.47 (1.14)	20.7*
LMIC		0.87 (1.22)	0.27 (0.74)	0.20 (0.39)	0.54 (1.78)	0.47 (1.75)	0.47 (1.13)	0.70 (2.71)	0.36 (1.39)	1.1
General workforce										
HIC	0.21 (0.29)	0.22 (0.34)	0.21 (0.34)	0.23 (0.33)	0.24 (0.45)	0.22 (0.41)	0.24 (0.39)	0.23 (0.39)	0.20 (0.42)	0.2
UMIC	0.09 (0.26)	0.16 (0.45)	0.14 (0.37)	0.21 (0.52)	0.29 (0.68)	0.23 (0.55)	0.33 (0.81)	0.34 (0.84)	0.33 (0.79)	16.7*
LMIC	0.41 (1.28)	0.28 (0.65)	0.18 (0.39)	0.35 (1.10)	0.47 (1.45)	0.45 (1.40)	0.49 (1.73)	0.58 (1.97)	0.52 (1.67)	9.2*
Healthcare occupation wage ratio (mean for women, mean for men)										
Support and allied healthcare										
HIC	0.97 (0.92,1.02)	0.94 (0.85,1.03)	0.94 (0.85,1.03)	0.94 (0.82,1.05)	0.92 (0.78,1.07)	0.90 (0.79,1.00)	0.92 (0.80,1.03)	0.98 (0.86,1.11)	0.87 (0.78,0.96)	− 0.6 (− 1.3, − 0.1)
UMIC	1.05 (0.97,1.13)	0.98 (0.98,0.98)	0.96 (1.01,0.91)	0.88 (0.85,0.90)	0.92 (0.74,1.10)	0.84 (0.75,0.95)	1.07 (0.81,1.33)	0.87 (0.78,0.95)	0.90 (0.70,1.10)	− 1.3 (− 4.2*, 1.0)
LMIC		0.85 (0.08,1.62)	0.39 (1.13,0.64)	0.40 (0.36,0.44)	0.34 (0.15,0.54)	0.78 (0.44,1.13)	0.51 (0.34,0.69)	0.66 (0.22,1.11)	0.46 (0.41,0.52)	− 1.4 (8.2, − 3.2)
Clinical and technical healthcare										
HIC	1.47 (1.13,1.83)	1.40 (1.18,1.62)	1.40 (1.12,1.68)	1.14 (0.86,1.43)	1.45 (0.93,1.98)	1.30 (0.92,1.68)	1.36 (0.93,1.79)	1.31 (1.10,1.52)	1.45 (1.26,1.63)	− 0.4 (− 0.1, − 0.6)
UMIC	1.32 (1.34,1.29)	1.34 (1.16,1.54)	1.28 (1.05,1.50)	1.23 (1.07,1.40)	1.15 (0.90,1.40)	0.94 (0.80,1.09)	1.07 (0.97,1.18)	1.14 (0.77,1.52)	1.22 (0.07,1.54)	− 2.9* (− 6.8*, − 0.2)
LMIC		0.70 (0.23,1.18)	0.40 (0.53,0.26)	0.42 (0.36,0.47)	0.76 (0.57,0.95)	0.76 (0.66,0.86)	0.70 (0.55,0.86)	0.65 (0.43,0.87)	0.74 (0.53,0.94)	5.4 (7.9, 7.6)
All healthcare										
HIC	1.11 (0.94,1.27)	1.03 (0.89,1.17)	0.04 (0.88,1.19)	1.00 (0.83,1.17)	0.04 (0.80,1.28)	1.01 (0.81,1.20)	1.03 (0.82,1.23)	1.04 (0.89,1.20)	0.96 (0.83,1.09)	− 0.9 (− 1.1, − 0.7)
UMIC	1.12 (1.07,1.18)	1.12 (1.03,1.21)	1.10 (1.03,1.17)	1.06 (0.92,1.19)	1.03 (0.79,1.26)	0.90 (0.76,1.03)	1.05 (0.86,1.25)	1.00 (0.78,1.22)	1.01 (0.70,1.32)	− 1.6 (− 5.0*, 0.8)
LMIC	0.65 (0.48,0.83)	0.77 (0.18,1.35)	0.41 (0.35,0.47)	0.41 (0.36,0.45)	0.54 (0.34,0.75)	0.75 (0.52,0.98)	0.59 (0.41,0.78)	0.64 (0.29,0.98)	0.56 (0.44,0.68)	0.3 (3.2, 0.0)

Abbreviations: *AAPC* average annual percentage change, *HIC* high-income country, *UMIC* upper-middle-income country, *LMIC* lower middle-income country

*Statistically significant AAPC result (*p* value less than 0.01)

### Gender ratio

In lower- and upper-middle-income countries, *gender ratios* in the general workforce increased between 2006 and 2014 (AAPC_LMIC_ 11.8%, *p* < 0.01; AAPC_UMIC_ 4.3%, *p* < 0.01) (Table 3). In high-income countries, gender ratios in the general workforce remained constant (AAPC_HIC_ 0%, *p* = 1.0). There was proportionally more women in the health workforce compared to the general workforce across lower-middle-, upper-middle- and high-income countries, and health workforce *gender ratios* increased between 2006 and 2014 (AAPC_LMIC_ 6.4%, *p* = 0.10; AAPC_UMIC_ 5.6%, *p* < 0.01; AAPC_HIC_ 1.1%, *p* < 0.01). In clinical and technical health occupations, the *gender ratio* was lower than the average health workforce across equivalent country income groups but remained higher than the general workforce between 2006 and 2014. Across each country income group, the *gender ratio* increased slightly over time, but this trend was only significant in upper-middle-income countries (AAPC_LMIC_ 4.5%, *p* = 0.30; AAPC_UMIC_ 5.8%, *p* < 0.01; AAPC_HIC_ 1.1%, *p* = 0.10).

**Fig. 3:**
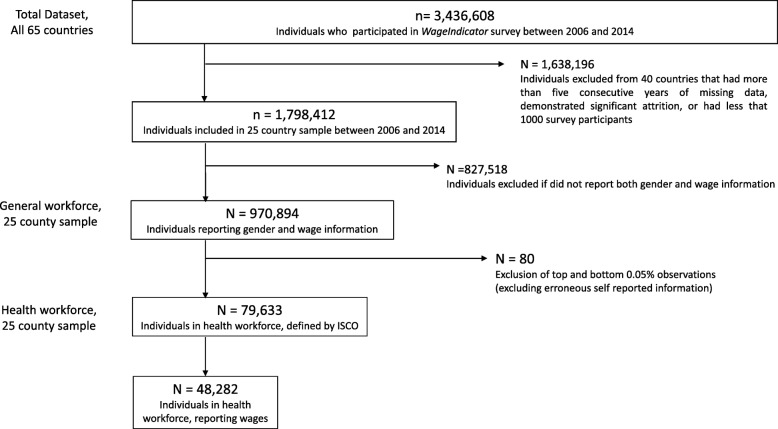
Gender ratios in the general workforce and health workforce (**a**) and in healthcare occupations (**b**). **a** Gender ratios in the general workforce and health workforce. **b** Gender ratios within healthcare (clinical/technical and allied/support occupational groupings)

In allied and support health occupations, the *gender ratio* over time across lower-middle-, upper-middle- and high-income countries was higher than in clinical and technical occupations and higher than in the general workforce (LMIC 0.50 to 0.66; UMIC 0.54 to 0.80; HIC 0.77 to 0.82). Across each country income group, the gender ratio increased over time and was significant in upper-middle- and high-income countries (AAPC_LMIC_ 2.8, *p* = 0.30; AAPC_UMIC_ 3.9, *p* < 0.01; AAPC_HIC_ 0.70, *p* < 0.01). In both clinical and allied healthcare occupation groups, the most striking increase in *gender ratios* occurred in upper-middle-income countries. Graphical illustrations of gender ratio time trends are shown in Fig. 3a, b.

### Gender wage gap

In the general workforce, the *gender wage gap* increased in lower- and upper-middle-income countries but remained relatively constant in high-income countries between 2006 and 2014 (AAPC_LMIC_ 9.2%, *p* < 0.01; AAPC_UMIC_ 16.7%, *p* < 0.01; AAPC_HIC_ 0.20, *p* = 0.80) (Table 3). In the health workforce, there was a significant increase in the *gender wage gap* in upper-middle-income countries (AAPC_UMIC_ 20.7%, *p* < 0.01) and insignificant changes in lower-middle- and high-income countries (AAPC_LMIC_ 1.1%, *p* = 0.90; AAPC_HIC_ 1.0%, *p* = 0.70).

**Fig. 4:**
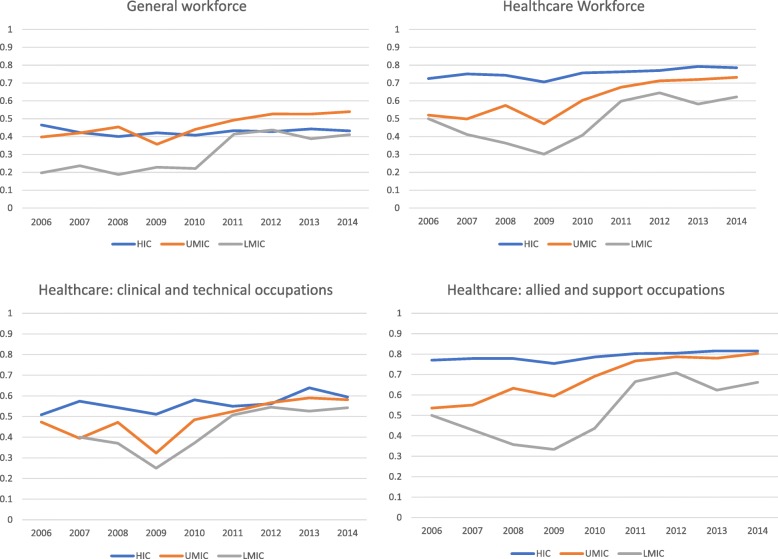
Gender wage gaps in the general workforce and health workforce (**a**) and gender wage gaps within healthcare (clinical/technical and allied/support occupational groupings) (**b**). **a** Gender wage gaps in the general workforce and health workforce. **b** Gender wage gaps within healthcare (clinical/technical and allied/support occupational groupings)

In clinical and technical occupations, the *gender wage gap* increased between 2006 and 2014 in lower- and upper-middle-income country groups (AAPC_LMIC_ 3.9%, *p* = 0.80; AAPC_UMIC_ 11.3%, *p* < 0.01), but declined in high-income countries (0.38 to 0.23; AAPC_HIC_ 0.6, *p* = 0.80). In allied and support occupations, the *gender wage gap* in high-income countries increased slightly between 2006 and 2014 (0.10 to 0.18), whereas the *gender wage gap* in upper-middle-income countries increased significantly (AAPC_UMIC_ 14.9%, *p* < 0.01). The *gender wage gap* in allied and support occupations in lower-middle-income countries was higher than other country groups (up to 0.95 in 2007) but was much more variable. Graphical illustrations of gender wage gap time trends are shown in Fig. 4a, b.

### Healthcare wage ratio

Healthcare wage conditions mostly declined between 2006 and 2014. In high-income countries, the overall *healthcare occupation wage ratio* declined from 1.11 in 2006 to 0.96 in 2014 (APCC − 0.9, *p* = 0.10) (Table 3). Health workers who were men earned higher on average than the general workforce (*healthcare occupation wage ratio* 1.27 to 1.09). Health workers who were women earned lower on average than the general workforce (*healthcare occupation wage ratio* 0.94 to 0.83). In clinical and technical occupations, the *healthcare occupation wage ratio* was higher than the general workforce (1.48 in 2006 and 1.44 in 2014; AAPC − 0.40, *p* = 0.70), but women’s *healthcare occupation wage ratio* was consistently lower than men’s (1.13 in 2006 and 1.25 in 2014 for women, compared to 1.82 in 2006 and 1.63 in 2014 for men). In allied and support occupations, the *healthcare occupation wage ratio* declined slightly over time (0.97 in 2006 and 0.87 in 2014; AAPC − 0.60%, *p* = 0.20), and men’s *healthcare occupation wage ratio* was consistently higher than women’s (1.02 to 1.11; compared 0.92 to 0.78).

In upper-middle-income countries, although there was a decline in wage conditions relative to the general workforce over time, there was a notable divergence between men’s and women’s *healthcare occupation wage ratios* in both clinical and allied healthcare occupation groups, over time. In the overall health workforce, the *healthcare occupation wage ratio* decreased from 1.12 in 2006 to 1.01 in 2014; this reflected a slight increase in men’s *healthcare occupation wage ratio* (AAPC + 0.80%, *p* = 0.40) and a significant decrease in women’s *healthcare occupation wage ratio* (− 5.0%, *p* < 0.01). In clinical and technical occupations, the overall healthcare occupation wage ratio decreased (AAPC − 2.9%, *p* < 0.01), driven by a decrease in the wage conditions of women (AAPC − 6.8%, *p* < 0.01). Men’s clinical *healthcare occupation wage ratio*, however, demonstrated an insignificant increase in this time period. In allied and support occupations, a similar pattern was observed: an overall decline from 1.05 in 2006 to 0.90 in 2014 (AAPC − 1.3%, *p* = 0.20), reflecting a divergence in wage conditions between men (AAPC + 1%, *p* = 0.60) and women (AAPC − 4.2%, *p* < 0.00).

In lower-middle-income countries, the *healthcare occupation wage ratio* pattern was more varied. Despite worse wage conditions relative to the general workforce overall, men’s *healthcare occupation wage ratio* was still consistently higher than that for women. In the overall health workforce, the *healthcare occupation wage ratio* shifted little from 0.65 in 2006 to 0.56 in 2014 (APCC 0.30%, *p* = 0.90; 0.83 to 0.68 for men, 0.48 to 0.44 for women). In clinical and technical occupations, the overall *healthcare occupation wage ratio* increased slightly from 0.70 in 2006 to 0.74 in 2014 (AAPC 5.4%, *p* = 0.20), which reflected discrepancies between men’s wage conditions (1.17 to 0.94) and women’s wage conditions (0.23 to 0.53). In allied and support occupations, wage conditions were variable but generally consistent with the pattern seen in clinical health occupations. The overall *healthcare occupation wage ratio* declined slightly from 0.65 in 2006 to 0.46 in 2014 (AAPC − 1.4%, *p* = 0.80), with men’s wage conditions higher than women’s (0.83 to 0.52 for men; 0.48 to 0.41 for women).

**Fig. 5:**
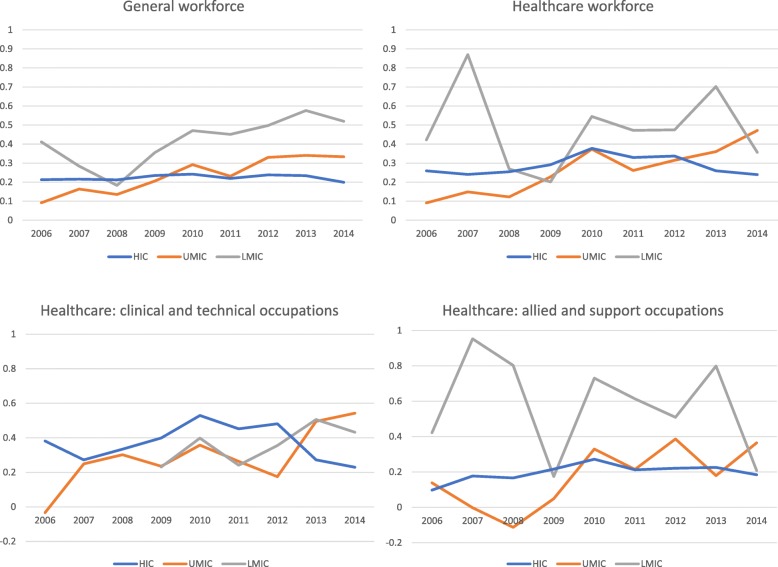
Wage conditions by gender and healthcare occupation group in high-income countries (**a**), upper-middle-income countries (**b**) and lower-middle-income countries (**c**). **a** Healthcare occupation wage ratio in high-income countries, by gender and healthcare occupation group. **b** Healthcare occupation wage ratio in upper-middle-income countries, by gender and healthcare occupation group. **c** Healthcare occupation wage ratio in lower-middle-income countries, by gender and healthcare occupation group

Figure 5a–c presents the *healthcare occupation wage ratio*, the ratio between reported healthcare salaries compared to the general workforce in high, upper-middle and lower-middle-income countries, by gender and healthcare occupation group.

## Discussion

We utilised *WageIndicator* data as an exploratory means to gain insight into health workforce participation and remuneration trends from a gender perspective, by calculating *gender ratio*, *gender wage gap* and *wage condition* trends in the general workforce and the health workforce over 25 countries between 2006 and 2014.

We found that the health workforce is feminising, particularly in lower-middle- and upper-middle-income countries. In our sample, the feminisation of the health workforce was driven largely by an increase in the proportion of women in allied and support professions in lower-middle- and upper-middle-income countries alongside a less-steep increase in the proportion of women in clinical and technical professions in all country groups. A significant increase in the health workforce gender ratio occurred in upper-middle-income countries, which may reflect the growth of the health sector as well greater opportunities for women to enter the health workforce. Gender trends in the health workforce mirrored general workforce trends, although there were proportionally more women in health occupations than in the general workforce in most years and country groups. This finding is consistent with current reports [49–51].

There was a substantial gender wage gap across the general and health workforce in all country groups. On average, women were paid 24 to 35% less than men in the general workforce, and 26 to 36% less than men in the health workforce. The gender wage gap was pronounced in all country and occupation groups, although the largest gender wage gaps were seen in allied and support occupations in lower-middle-income countries. Whilst the gender wage gap remained constant in high-income countries, the gap was increasing over time in lower- and upper-middle-income countries.

Increasing proportions of women in the health workforce was also associated with a decrease in wage conditions over time relative to the general workforce. This is consistent with the cross-sectional analysis reported by Tidjens et al. who reported that increasing proportions of women over selected health occupations were associated with decreasing wage rank [22]. Although health workforce wage conditions deteriorated in most country groups, women’s wage conditions were consistently worse than men’s. Women’s wage disadvantage was most pronounced in clinical and technical occupations across upper-middle- and high-income countries, and in allied and support occupations in lower-middle-income countries. This may represent—in addition to the overall maturity of the health system—a lag-time between the feminisation of a particular health occupational group and, subsequently, how these occupations adjust to ensure gender equitable salaries.

These exploratory results, if a true reflection of the global health workforce, have significant implications for health policy and planning, and specifically for the development, organisation and management of human resources for health. They also point to the need for a more nuanced approach to health workforce planning that considers national levels of development, focuses on specific health occupations including vertical and horizontal occupational segregation and takes an explicit gendered approach to analysis.

### Macroeconomics and feminism: health workforce trends from a gender perspective

In the context of an expanding [52, 53] and simultaneously feminising [51] global health workforce, our results suggest that as more women enter a professional group, gender wage differences increase and women’s wage conditions decrease relative to the general workforce. This finding was particularly pronounced in lower-middle- and upper-middle-income countries, where increasing proportions of women employed in healthcare between 2006 and 2014 were associated with widening gender wage gap and overall deterioration of wage conditions. This pattern reflects broader societal gender stereotypes introduced above, which tend to associate women with—often unpaid—caregiving work [32–34].

An expanding health workforce, necessary to sustain health systems and reach UHC targets, may confront financing challenges such as public health expenditure caps or wage bill ceilings [16, 50, 54]. As women in the health workforce receive relatively lower wages for similar work, they appear to do “more for less”. This establishes a perverse economic incentive whereby increasing the number of women in the health workforce may be a “good buy”, keeping the overall health wage bill down. Unless we dissect this trend with a feminist or gender lens, this tension may not be recognised.

Feminist economists have long argued that markets are socially embedded and therefore gendered social systems [55]. The feminisation of the health workforce is evidence of how wage inequality is not just about fairness, but leads to both unequal power and unequal opportunities for women [56, 57]. A feminised health workforce with increasingly lower wages leaves the disproportionate number of women working in health with less economic power. It also changes the status of the health section in society, further devaluing care work economically and emphasising the social norm that care work is women’s work. Ensuring that women have equal pay for equal work is necessary but insufficient to address these wider concerns.

### Limitations

*WageIndicator* data facilitated an exploratory analysis of health workforce trends using a gender lens. This dataset was unique, in that it provided information on the gender composition of the health workforce as well as self-reported wage data trends, to a level of detail that other surveys had not. Despite this, the approach was limited in a number of ways.

First, use of web survey data such as *WageIndicator* is not without its challenges. Despite the ability to collect data in a low-cost, rapid and continuous manner, web surveys are limited by the representativeness of the collected data with respect to the population of interest [22, 40]. Due to the lack of a sampling frame, web survey data reports information from a specific subpopulation: those with internet access, visiting the specific website, and who chose to complete the survey. Thus, web surveys are susceptible to self-selection and reporting bias [40]; thus, the representativeness of *WageIndicator* data may be limited [22, 40].

Second, the quality of data was insufficient to allow for further detailed analysis. Due to small numbers in particular groups, we were unable to disaggregate further following initial decomposition by country income group, occupational group and time. This meant that we were unable to explore national trends or demographic trends over time. We were also unable to explore horizontal differences in the gender composition of specific occupations over time. For the same reason, we were unable to perform advanced statistical analyses on our sample. We present simple trend analyses as a starting point, with the recognition that more comprehensive time panel data may, in the future, yield more accurate results.

Third, we recognise the complex nature of gender norms and dynamics—including the spectrum of gender identifies and their intersection with other social factors—and that we were only able to look at a small aspect of a more complex whole in relations to gender and power, and how these operate in the health workforce. Furthermore, we also note the interaction between gender (social) and sex (biological and physical characteristics). Whilst some biological considerations do shape women’s and men’s careers, we align our work with the prevailing view [25, 27, 35, 51], that it is the gendered nature of the health workforce that treats biological functions unfairly.

Fourth, in this survey, we were unable to quantify unpaid labour, such as caregiving. Women are known to comprise the majority of unpaid health workers [13, 23, 58]. By failing to recognise the unpaid health workforce, we further silence the voices of those—mainly women—who are not a part of the formal health economy. Furthermore, we tend to define occupations in fixed categories which may not capture the multiple or blurred roles women occupy in the health and care economy [13]. Although we were not able to address these limitations in the body of the research, we do recognise this as a necessary area of research and policy development.

Despite these limitations, our findings are consistent with current literature reporting participation rates and wage inequalities between women and men in global health [13, 51, 59]. Data limitations have plagued health workforce research, especially in low- and middle-income countries [16]. Sourcing accurate wage information is difficult; even ILO wage estimates must sometimes rely on self-reported information derived from household surveys [60]. So, whilst *WageIndicator* survey data is imperfect, it is a novel way of gaining insights into health workforce dynamics from a gender perspective in the absence of comprehensive and sufficiently disaggregated data. Given these limitations, the Health Workforce Department of the WHO, in collaboration with the ILO, is currently compiling gender disaggregated wage data and has plans to publish and make public their findings [61].

### Looking forward

The call for UHC has significant implications for health workforce policy and planning. Modelled estimates predict a shortfall of around 18 million health workers needed to meet health system needs [50]. Dealing with an expanding health workforce is difficult, but sustainable financing for health workers is achievable in most low-income- and lower-middle-income countries through progressive fiscal policies and reprioritisation of domestic expenditure [17]. Health worker wages comprise a major proportion of public health expenditure and must be of central concern when planning to realise the objective of “health for all” [16, 17]. However, this analysis cannot be gender-blind, because a feminising health workforce has implications for wages which translates into significant ramifications for national health financing policy.

The exploration of trends over national levels of economic development (reflecting resource constraints of national health systems) and over healthcare occupational groupings (reflecting historical gender trends) may offer clues to help shape health policy that addresses the gendered nature of health workforce participation and remuneration in order to transform gender inequities. The dramatic increase in the proportion of women employed in the healthcare sector, particularly in lower- and upper-middle-income countries, offers an opportunity to improve national and global standards: for example, we know that gender equality in the workforce offers significant economic gains [62] and that investing in health workers leads to economic growth [52]. We also know that macroeconomic gains are possible when women are able to develop their full labour market potential [63]. So, promoting a gender equitable health workforce constitutes a substantial investment in national economic and social prosperity.

### Conclusion

In this study, we have attempted to explore gender trends in the health workforce in multiple countries over time and its implication on wage conditions in the health workforce, using *WageIndicator* data. Our approach combines a descriptive analysis of gender trends in participation and remuneration in the health workforce over time, with a conceptual discussion on the gender implications of our results from macroeconomic and feminist perspectives. Our findings suggest that the health workforce is feminising, that women are paid less than men for the same work and that the gender wage gap is increasing, especially in lower- and upper-middle-income countries. In order for future health workforce policy and planning to be as effective and equitable as possible, we highlight the need for a high-level discussion on gender dynamics and the global health workforce that combines economics and critical feminist analysis.

## Research in context

### Evidence before this study

Although investing in human resources for health is an international priority, gender has been a missing dimension of policy discussions. A possible reason for this evidence gap is the lack of internationally comparable wage data that are gender-disaggregated and contain sufficiently detailed information about health sector occupations and their corresponding wages over time. Because of these limitations, critical, evidence-based discussions about gender dynamics in the global health workforce and whether these trends affect wage conditions have been limited.

### Added value of this study

This study uses a novel approach of online wage survey data to interrogate the feminisation of the global health workforce and its impact on wage conditions globally. It is the first to our knowledge that attempts to explore this association. Our approach brings together a descriptive analysis of gender trends in the global health workforce (participation and remuneration) over time with a feminist critique.

### Implications of all the available evidence

1. The health workforce is feminising, notably in lower-middle income and upper-middle-income countries
2. Women are paid less than men in the health workforce
3. The gender wage gap is increasing especially in lower- and upper-middle-income countries
4. Increasing female participation in the health workforce is associated with decreasing wage conditions relative to the general workforce; women are disproportionately disadvantaged in wage conditions relative to men in all country groups and healthcare occupations.
5. There is a perverse economic incentive to have a feminised workforce because it appears that women will “do more for less” and this must be a consideration of health workforce and health system financing policy discussions at national and international levels
6. Feminist analysis sheds new light on economics-based health workforce policy discussions

## Data Availability

We used data from the *WageIndicator* questionnaire, which is posted continuously at all national WageIndicator websites (http://www.wageindicator.org) We were granted access to data for free for the purpose of academic research from the IZA, Germany, at http://idsc.iza.org/?page=27&stid=1025. All data generated or analysed during this study are included in supplementary information files.

## Abbreviations

EAPEP: Economically Active Population Estimates and Projections
GNI: Gross national income
ILO: International Labour Organization
ISCED: International Standard Classification of Education
ISCO-08: International Standard Classification of Occupations, 2008 revision
IZA: Institute of Labor Economics
OECD: Organisation for Economic Cooperation and Development
PPP: Purchasing power parity
SDG: Sustainable Development Goals
UHC: Universal Health Coverage
UK: United Kingdom
US: United States
WHO: World Health Organization
