# Correction to: Feminisation of the health workforce and wage conditions of health professions: an exploratory analysis

**DOI:** 10.1186/s12960-019-0425-x

**Published:** 2019-11-15

**Authors:** Geordan Shannon, Nicole Minckas, Des Tan, Hassan Haghparast-Bidgoli, Neha Batura, Jenevieve Mannell

**Affiliations:** 1 Centre for Gender and Global Health, Institute for Global Health, University College London, 3rd floor, Institute of Child Health, 30 Guilford Street, London, WC1N 1EH UK; 2 STEMA, Institute for Global Health, University College London, 3rd floor, Institute of Child Health, 30 Guilford Street, London, WC1N 1EH UK; 3 Centre for Global Health Economics, Institute for Global Health, University College London, 3rd floor, Institute of Child Health, 30 Guilford Street, London, WC1N 1EH UK

**Correction to: Hum Resour Health (2019) 17:72**

**https://doi.org/10.1186/s12960-019-0406-0**

The original article [1] contained an error in the presentation of all figures and tables; each figure and table is now set out and designated appropriately in the original article.
